# C-C Coupling Reactions between Benzofurazan Derivatives and 1,3-Diaminobenzenes

**DOI:** 10.3390/molecules22050684

**Published:** 2017-04-26

**Authors:** Gabriele Micheletti, Silvia Bordoni, Elena Chugunova, Carla Boga

**Affiliations:** 1 Department of Industrial Chemistry ‘Toso Montanari’, Alma Mater Studiorum Università di Bologna Viale Del Risorgimento, Bologna 4402136, Italy; Silvia.bordoni@unibo.it; 2 A.E. Arbuzov Institute of Organic and Physical Chemistry, Kazan Scientific Center, Russian Academy of Sciences, Akad. Arbuzov st. 8, Kazan, Tatarstan 420088, Russia; chugunova.e.a@gmail.com

**Keywords:** benzofurazans, aromatic substitution, 1,3-diaminobenzenes

## Abstract

Aromatic substitution reactions between 1,3-diaminobenzene and chloronitrobenzofurazan derivatives have never been reported so far. The aim of the current study was to synthesize novel electron-donor and -acceptor architectures of interest in applied fields and to provide new insights on the nucleophilic behavior of 1,3-diaminobenzenes. The reaction of 1,3-dipiperidinyl-, 1,3-dimorpholinyl-, 1,3-dipyrrolidinyl-, or 1,3-dimethylamino-benzene with 7-chloro-4,6-dinitrobenzofuroxan or with a series of chloro-nitrobenzofurazans has been carried out in mild conditions. The partners reactivity has been investigated by monitoring the reaction course through ^1^H-NMR spectroscopy. The reaction occurred in a regioselective way, providing in good yields the novel C-C coupling compounds. Indications on the reactivity behavior for the studied nucleophiles have been relieved.

## 1. Introduction

**Scheme 1:**
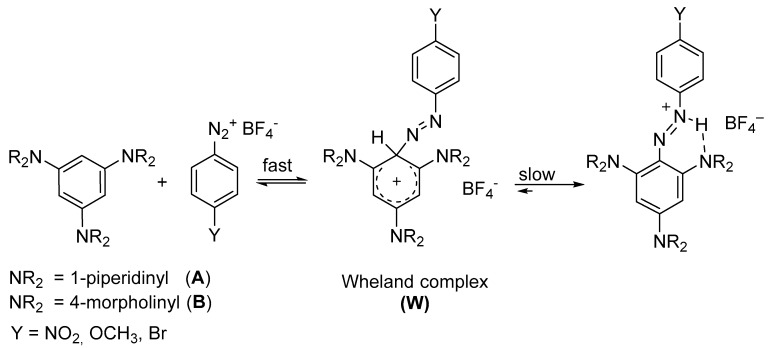
Simplified reaction pathway of the azo coupling reaction.

The aromatic substitution is one of the most exploited reactions in organic chemistry. The reaction can simply be indicated as aromatic substitution if both the reagents are aromatic, whereas the distinction into S_E_Ar and S_N_Ar has been invoked when only one reagent is aromatic. The case of C-C coupling between neutral aromatic substrates requires opposite features in the reagents (i.e., electron-rich vs. electron-poor). The 1,3,5-triaminobenzene derivatives—first studied by the pioneer work of Effenberger [1]—belong to neutral electron-rich aromatic substrates and are able to react at the neutral carbon atom [1,2]. (These kinds of nucleophiles have been reported to react with a plethora of electrophiles, such as proton [1,2,3,4,5,6,7,8,9,10], halogens [11,12], acyl- [13,14,15], alkyl- [16], and aryl-halides [17]. The coupling between 1,3,5-triaminobenzene of types **A** and **B** and aryl diazonium salts gave stable Wheland intermediates (**W**) by the azo-coupling reaction [18] (Scheme 1), conversely to what is usually reported in the textbooks. They provided evidence of the reversibility [19], and the proton departure from Wheland intermediate as the rate-determining step [20].

**Scheme 2:**
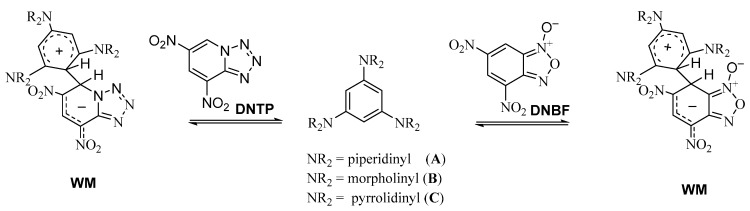
The first examples of detection and characterization of Wheland-Meisenheimer (**WM**) intermediates. DNBF: 4,6-dinitrobenzofuroxan; DNTP: 4,6-dinitrotetrazolopyridine.

The coupling of 1,3,5-triaminobenzenes **A**–**C** (Scheme 2) with 4,6-dinitrobenzofuroxan (**DNBF**) provided the first example of characterized Wheland-Meisenheimer (**WM**) intermediate. The zwitterionic nature is generated by the contemporary presence of Wheland on the nucleophilic fragment and Meisenheimer on the electrophilic one. Further, **WM** intermediates have been identified by the sym-triaminobenzenes with 4,6-dinitrotetrazolopyridine (**DNTP**) or 2,3,4-trinitrothiophene [21]. 4,6-Dinitrobenzofuroxan (**DNBF**) and 4,6-dinitrotetrazolopyridine (**DNTP**) are 10π electron-deficient heteroaromatics exhibiting reactivity behavior to be ranked as “superelectrophilic heteroaromatics” [22,23]. Their Mayr electrophilicity values [24,25,26,27,28] are −5.06 [29] and −4.67, [22], respectively.

Finally, **DNBF** and **DNTP** gave **WM** intermediates by C-C coupling with other nucleophilic substrates, such as 2-aminothiazole [30] or 2,4-di(pyrrolidinyl)thiazole [31]. Further, the C-C coupling between a series of chloro-nitrobenzofurazans and sym-triaminobenzenes gave access to highly conjugated structures of interest in the material field [32]. In the study, the first Wheland intermediate in benzofurazan series was isolated and fully characterized [32]. Among the mechanistic studies here described, it is noteworthy that benzofuroxans and benzofurazans are interesting reagents from a biological point of view—for example, as fluorescent probes [33,34] or fungicides [35]. Nitric oxide constitutes a biologically important molecule of the endothelium-derived relaxing factor, and the benzofuroxan moiety is well known for its ability to release NO under physiological conditions [36,37]. Several derivatives obtained from the combination of a benzofuroxanyl moiety with distinct bioactive substructure have recently received particular attention as anti-inflammatory [38] or antimicrobial agents [39]. Moreover, nitrobenzofurazans and nitrobenzofuroxans are also recognized to be versatile compounds in optoelectronic, agrochemical, or material fields [40,41].

Contrarily to the case of 1,3,5-triaminobenzenes, few examples of C-C coupling involving diaminobenzene derivatives [17,42,43,44] are present in the literature, likely due to their minor carbon nucleophilicity with respect to 1,3,5-triaminobenzenes.

Scarce literature reports on the reactivity of 1,3-diaminobenzenes suggested that we react them with 7-chloro-4,6-dinitrobenzofuroxan and chloro-nitrobenzofurazan derivatives. We investigated the reactivity of these nucleophiles to achieve compounds of interest for applications related to the simultaneous presence of electron-poor and electron-rich functions on the same scaffold.

## 2. Results and Discussion

**Scheme 3:**
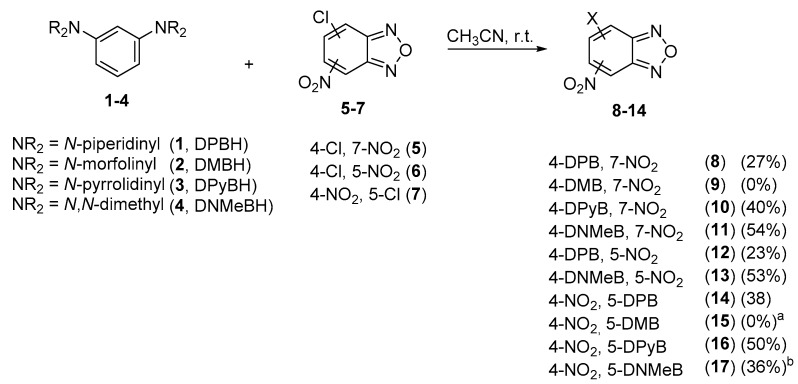
Combination between nucleophiles **1**–**4** and electrophiles **5**–**7**. ^a^ Reaction run at 80 °C also with no conversion improving. ^b^ In the presence of alumina, the yield became 60%. DPBH: 1,3-bis(*N*-piperidinyl)benzene, DMBH: 1,3-bis(*N*-morpholinyl)benzene, DPyBH: 1,3-bis(*N*-pyrrolidinyl)benzene, DNMeBH: 1,3-bis(dimethylamino)benzene; DPB: 1,3-bis(*N*-piperidinyl)phenyl; DMB: 1,3-bis(*N*-morpholinyl)phenyl; DPyB: 1,3-bis(*N*-pyrrolidinyl)phenyl; DNMeB: 1,3-bis(dimethylamino)phenyl.

The reaction between 1,3-bis(*N*,*N*-dialkylamino)benzenes **1**–**4** (indicated thereafter by the acronyms shown in Scheme 3) and the chloro-nitrobenzofurazans **5**–**7** was carried out in acetonitrile at room temperature with an equimolar amount of reagents.

**Scheme 4:**
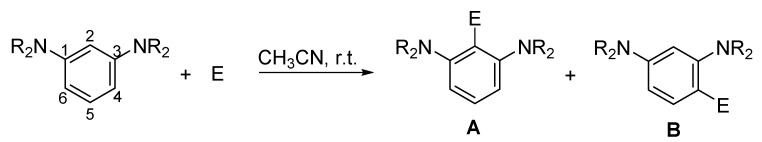
Possible reaction products derived from the reaction of 1,3-diaminobenzene derivatives with electrophilic reagents.

Two distinct isomers (**A** and **B** in Scheme 4) might be expected from the attack of the bis(dialkyl)aminobenzene (Scheme 4) at the carbon atom in position 2 or 4. As a matter of fact, the isomer **B** has solely been formed. By preventing the nucleophilic attack in position 2, steric hindrance did not likely permit the formation of isomer **A**.

The C-C coupling products were purified by flash chromatography (FC) on silica gel column and were fully characterized. In the cases of the reactions involving 1,3-bis(*N*-morpholinyl)benzene (DMBH, **2**), no product was formed, even by increasing the reaction temperature within 80 °C. Yields shown in Scheme 3 were calculated without considering that a half equivalent of the nucleophile might react with the hydrochloric acid formed as reaction co-product. This salification may be responsible for decreasing the nucleophilic efficiency so that a 50% row-yield was the maximum expected. Conversely, the achieved 54%/53% yields after work-up process for compounds **11**/**13**, respectively, suggests that protonation on the reaction products might occur, as previously observed in the case of triaminobenzenes with benzofurazans **5**–**7** [32]. Occasionally, to remove the formed HCl from the reaction mixture, some experiments were run in the presence of basic alumina. Neutralization procedure increased the yield in few cases, probably ascribed to the disrupting interactions with the Al_2_O_3_ stationary phase.

**Scheme 5:**
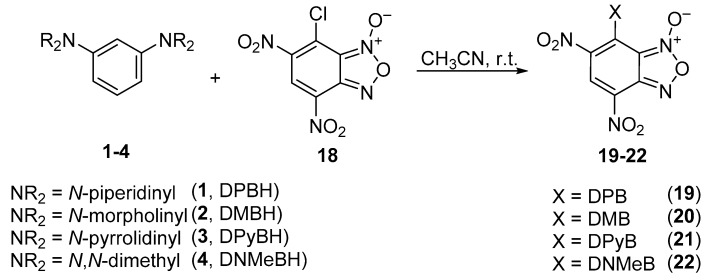
Reaction between **18** and 1,3-diaminobenzene derivatives **1**–**4**.

By testing the reactivity of **1**–**4** with the purpose of using more efficient electrophiles, we decided to use 7-chloro-4,6-dinitrobenzofuroxan (**18**), the electrophilicity of which is enhanced by introducing a further nitro and an N-oxide function in the oxadiazole ring (Scheme 5). In the latter case, the conversion was consistently getting raised so that the reaction on 1,3-di(morpholinyl)benzene (**2**) afforded derivative **20**.

Once the products between **1**–**4** and **5**–**8** were isolated and fully characterized, we planned to investigate the reaction course. For this purpose, an equimolar amount of each electrophile/nucleophile couple—dissolved in CDCl_3_ or CD_3_CN—was added, and the reaction was monitored within 72 h into the NMR tube. The results are reported in Table 1.

To analyze the solvent influence, we investigated reactions of **5** with nucleophiles **1** and **4** both in CDCl_3_ and CD_3_CN (Table 1, entries 1–4). Noticeably, the yields of the expected products raised up to 50% by using CD_3_CN solvent in all the reactions monitored. Quantitative yield was afforded in the case of electrophile **18**. The behavior is ascribed to the salification of the final product, as reported above for compounds **11** and **13**.

The conversion consistently increases by using dinitrobenzofuroxan as reagent. The data inferred by comparing (Table 1) the reaction of substrate **5** with nucleophile **1** or **3** (entries 2, 5) and reaction of **18** with nucleophile **1** or **3** (entries 9, 12) were expected on the basis of electrophilicity increasing due to the introduction of a further nitro group in the substrate. It has been reported that upon going from benzofurazan to benzofuroxan, the influence of the *N*-oxide in the pentatomic heteroaromatic ring was almost negligible [45,46].

By comparing the reaction data between **5** and the nucleophiles **1**, **3**, and **4** after 10 min (time within the salification process of the starting nucleophile and/or the reaction product can be considered negligible), the conversion relative on the nucleophilic species is DPBH < DNMeBH < DPyBH. Since the nucleophilicity of compounds **1**–**4** have not been reported so far, the above trend can be correlated to the nucleophilicity values of the substituent, in agreement with the equation developed by Mayr: *N*_Mayr_ = 15.65, 17.35, and 18.64 for morpholine, piperidine, and pyrrolidine [47], and 17.96 for dimethylamine [48], respectively. Similar considerations can be made by analyzing the reactions of **1** and **3** with **7** (entries 6, 8) and reactions of **1**, **2**, and **3** with **18** (entries 9, 10, 12). The reaction conversions of **7**, **18** with substrate **4** resulted lower or comparable with respect to the reaction with **1** or **2**; this observation might be explained by considering two distinct factors: steric hindrance due to both the proximity (*ortho*-position) of the nitro groups in the electrophile, and that of the dimethylamino group on the incoming nucleophile.

**Scheme 6:**
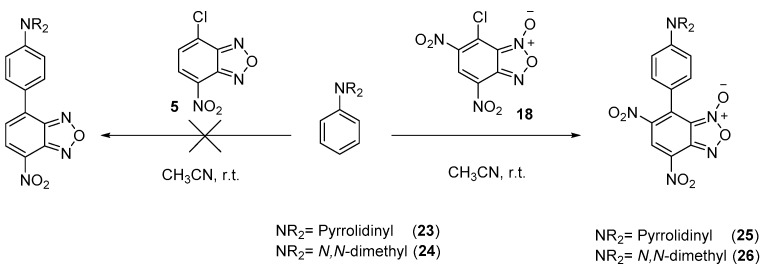
Reaction between **5** and **18** and monoaminobenzenes **23**–**24**.

To confirm this hypothesis and also to extend the study to the reactivity of monoaminobenzene derivatives, we reacted the chloro-nitrobenzofurazan **5** and the chlorodinitrobenzofuroxan **18** with *N*-pyrrolidinylbenzene (**23**) and *N*,*N*-dimethylaminobenzene (**24**) (Scheme 6) in equimolar amounts, and monitored the reaction course via by ^1^H-NMR spectroscopy.

We found that **5** did not react with either **23** or **24**, whereas the reactions with **18** occurred at room temperature to afford the C-C coupling in *para*-position with respect to the amino group of the nucleophile. Specifically, the ^1^H-NMR spectrum showed a 44% and 23% conversion, respectively, for **23** and **24** after 10 min. These findings are in agreement with the nucleophilicity values of the amino substituent reported above, and support the occurrence of steric hindrance to explain the unexpected very low conversion to **22** (Table 1, entry 11). The absence of the crowding in monoaminobenzenes permitted the formation of products **25** (from **18** and **23**) and **26** (from **18** and **24**), and thus gave a further confirmation of the increase in the reactivity of the electrophile ascribed to the presence of an additional nitro group.

**Scheme 7:**
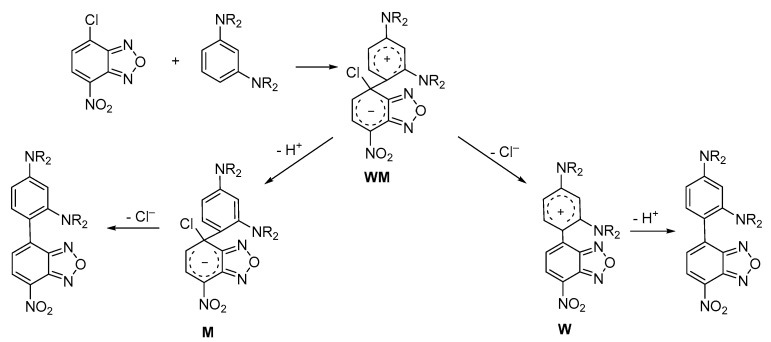
Possible intermediates in the S_E_Ar/S_N_Ar reaction between chloro-nitrobenzofurazans and 1,3-diaminobenzene derivatives.

Finally, the proposed mechanism for the current S_E_Ar/S_N_Ar reactions (shown in Scheme 7 for compound **5**) implies the formation of **WM** zwitterionic intermediate. The intermediate might evolve into a cationic Wheland-like intermediate (**W**) through the chloride displacement or to an anionic Meisenheimer like (**M**) intermediate via proton expulsion. The final substitution product can be generated from both intermediates. Due to presence of a chloride leaving group, the σ-anionic intermediate **M** (as well as **WM**) is an elusive species, which to the best of our knowledge has never been detected. The presence of electron-donor groups on the nucleophilic fragment might be responsible for the stabilization of **W** intermediate to let it be detectable. In the case of reaction between **5** and 1,3,5-tris(pyrrolidinyl)benzene [32], we already reported the isolation and characterization of **W**-type intermediate. The efforts to analogously trap elusive species in the NMR tube at low temperature for the reactions reported in Scheme 7 did not afford analogous results, indicating that the electronic features of two amino groups are not sufficient requisites to stabilize the σ-cationic intermediate.

## 3. Materials and Methods

### 3.1. General Methods

The ^1^H spectra were recorded with a Mercury 400 (Varian, Palo Alto, CA, USA) spectrometer operating at 400 MHz for ^1^H-NMR and 100.56 MHz for ^13^C-NMR. Signal multiplicities were established by Distortioless Enhanced by Polarization Transfer (DEPT) experiments. Chemical shifts were measured in δ (ppm) with reference to the solvent (δ = 7.26 ppm and 77.00 ppm for CDCl_3_, for ^1^H-, and ^13^C-NMR, respectively). *J* values are given in Hz. Electron spray ionization mass spectra (ESI-MS) were recorded with a WATERS 2Q 4000 instrument (Waters Corporation, Milford, MA, USA). IR spectra were recorded on a Perkin Elmer FT-IR spectrometer (Perkin Elmer, Waltham, MA, USA) Spectrum Two equipped with a UATR TWO ATR accessory (diamond crystal, DTGS detector; spectral resolution 4 cm^−1^). Chromatographic purifications (FC) were carried out on glass columns packed with silica gel (Merck grade 9385, 230–400 mesh particle size, 60 Å pore size) at medium pressure. Thin layer chromatography (TLC) was performed on silica gel 60 F254 coated aluminum foils (Fluka Chemie GmbH, Buchs, Switzerland).

### 3.2. Synthesis of 1,3-Di(piperidin-1-yl)benzene *(**1**)* and N^1^,N^1^,N^3^,N^3^-Tetramethylbenzene-1,3-diamine *(**4**)*

To a solution of 1,3-dichlorobenzene (0.85 mL, 7.45 × 10^−3^ mol) and amine (piperidine or dimethylamine 0.06 mol) in 50 mL of anhydrous THF, under nitrogen atmosphere, phenyl lithium (30 mL, 0.06 mol) was added dropwise. The mixture was stirred for 24 h at room temperature, then was quenched with water (50 mL). The organic layer was separated, and the aqueous fraction was extracted with diethyl ether (3 × 30 mL). The organic layer was anhydrified over magnesium sulfate and filtered. The product was purified by column chromatography on silica gel (*n*-hexane/ethyl acetate 2:1). The chemico-physical data of **1** [49,50] and **4** [51] are in good agreement with those reported in the literature.

### 3.3. Synthesis of 1,3-Dimorpholinobenzene *(**2**)* and 1,3-Di(pyrrolidin-1-yl)benzene *(**3**)*

The reaction was carried out in autoclave. Amine (morpholine or pyrrolidine, 0.07 mol) and potassium *tert*-butylate (5.4 g, 0.048 mol) were added to the solution of 1,3-dichlorobenzene (1.37 mL, 0.011 mol) in toluene (10 mL). The reaction was left at 160 °C for 4 days under magnetic stirring, then it was allowed to stand at room temperature and quenched with water (50 mL). The organic layer was separated, and the aqueous fraction was extracted with dichloromethane (3 × 30 mL). The organic layer was dried over anhydrous magnesium sulfate. The product was purified by column chromatography on silica gel. The chemico-physical data of **2** [49,50] and **3** [52] are in good agreement with those reported in the literature.

### 3.4. Reactions between (N,N-Dialkyl)-diaminobenzenes *(**1**–**4**)* and Compounds ***5**–**7***—General Procedure

To a stirred solution of (*N*,*N*-dialkyl)-diaminobenzene (0.1 mmol) in acetonitrile (5 mL), an equimolar amount of electrophile was added. The reaction was left at room temperature under magnetic stirring for about 12 h. The product was purified by column chromatography on silica gel. An analogous procedure but with a 2/1 nucleophile/electrophile relative molar ratio was used for the reactions of **18** with **5**–**7**, **23**, **24**; reactions of **5** with **23** and **24** did not work.

*4-(2,4-Di(piperidin-1-yl)phenyl)-7-nitrobenzo[c][1,2,5]oxadiazole* (**8**): eluent: *n*-hexane/ethyl acetate 8/2; yield 27%; brown solid, m.p.: >200 °C (dec); IR (*v*, cm^−1^): 1601, 1503, 1299, 1235; ^1^H-NMR (CDCl_3_, 400 MHz, 25 °C): δ (ppm): 8.52 (d, *J* = 8.1 Hz, 1H); 8.26 (br.s, 1H); 7.66 (d, *J* = 8.1 Hz, 1H); 6.64 (br.s, 2H); 3.35 (br.s, 4H); 2.85 (br.s, 4H); 1.85–1.60 (m, 6H); 1.46 (br.s, 6H);^13^C-NMR (CDCl_3_: 100.56 MHz, 25 °C): δ (ppm): 154.5; 154.0; 150.1; 143.5; 139.5; 133.7; 131.3; 125.8; 116.8; 109.0; 105.8; 53.4; 49.1; 25.9; 25.5; 23.9; ESI-MS (*m*/*z*): 408 [M + H]^+^, 430 [M + Na]^+^, 446 [M + K]^+^; HRMS (ES^+^) *m*/*z*: [M + H]^+^ calculated for C_22_H_26_N_5_O_3_ 408.2036 found 408.2038. 

*4-(2,4-Di(pyrrolidin-1-yl)phenyl)-7-nitrobenzo[c][1,2,5]oxadiazole* (**10**): eluent: petroleum light/diethyl ether 1/1; yield 40%; brown solid, m.p.: >280 °C (dec); IR (*v*, cm^−1^): 1605, 1499, 1288, 1235; ^1^H-NMR (CDCl_3_, 400 MHz, 25 °C): δ (ppm): 8.49 (d, *J* = 8.2 Hz, 1H); 7.72 (d, *J* = 8.9 Hz, 1H); 7.25 (d, *J* = 8.3 Hz, 1H); 6.27 (dd, *J*_1_ = 9.0 Hz, *J*_2_ = 2.2 Hz, 1H); 6.10 (s, 1H), 3.41 (t, *J* = 6.7 Hz, 4H), 3.06 (t, *J* = 6.7 Hz, 4H); 2.10–2.03 (m, 4H); 1.89–1.81 (m, 4H); ^13^C-NMR (CDCl_3_: 100.56 MHz, 25 °C): δ (ppm): 150.4; 150.1; 149.9; 143.6; 140.5; 134.7; 132.0; 130.9; 124.1; 111.3; 104.7; 97.3; 52.2; 48.0; 25.7; 25.4; ESI-MS (*m*/*z*): 380 [M + H]^+^, 402 [M + Na] ^+^, 418 [M + K]^+^; HRMS (ES^+^) *m*/*z*: [M + H]^+^ calculated for C_20_H_22_N_5_O_3_ 380.1723 found 380.1724.

*N^1^,N^1^,N^3^,N^3^-Tetramethyl-4-(7-nitrobenzo[c][1,2,5]oxadiazol-4-yl)benzene-1,3diamine* (**11**): eluent: petroleum light/diethyl ether 4/6; yield 54%; brown solid, m.p.: >280 °C (dec); IR (*v*, cm^−1^): 1600, 1478, 1297, 1235; ^1^H-NMR (CDCl_3_, 400 MHz, 25 °C): δ (ppm): 8.51 (d, *J* = 8.0 Hz, 1H); 8.01 (d, *J* = 7.6 Hz, 1H); 7.73 (d, *J* = 8.6 Hz, 1H); 6.49 (d, *J* = 8.6 Hz, 1H); 6.43 (s, 1H); 3.09 (s, 6H); 2.68 (s, 3H); ^13^C-NMR (CDCl_3_: 100.56 MHz, 25 °C): δ (ppm): 153.6; 152.2; 150.1; 143.7; 139.6; 134.4; 132.5; 131.8; 125.2; 114.9; 106.6; 102.6; 43.9; 40.7; ESI-MS (*m*/*z*): 328 [M + H]^+^, 350 [M + Na]^+^, 366 [M + K]^+^; HRMS (ES^+^) *m*/*z*: [M + H]^+^ calculated for C_16_H_18_N_5_O_3_ 328.1410 found 328.1413.

*4-(2,4-Di(piperidin-1-yl)phenyl)-5-nitrobenzo[c][1,2,5]oxadiazole* (**12**): eluent: petroleum light/diethyl ether 9/1; yield 23%; dark blue solid, m.p.: >80 °C (dec.); IR (*v*, cm^−1^): 1598, 1502, 1285, 1234; ^1^H-NMR (CDCl_3_, 400 MHz, 25 °C): δ (ppm): 7.91 (d, *J* = 9.5 Hz, 1H); 7.82 (d, *J* = 9.5 Hz, 1H); 7.42 (d, *J* = 8.4 Hz, 1H); 6.85-6.54 (m, 2H); 3.31 (br.s. 4H); 2.70–2.53 (m, 4H); 1.9–1.59 (m, 8H); 1.35 (m, 4H); ^13^C-NMR (CDCl_3_: 100.56 MHz, 25 °C): δ (ppm): 153.9; 150.7; 149.2; 146.3; 131.7; 128.0; 126.7; 114.5; 109.7; 107.4; 53.6; 49.4; 25.8; 25.5; 24.8; ESI-MS (*m*/*z*): 408 [M + H]^+^, 430 [M + Na]^+^, 446 [M + K]^+^; HRMS (ES^+^) *m*/*z*: [M + H]^+^ calculated for C_22_H_26_N_5_O_3_ 408.2036 found 408.2037.

*N^1^,N^1^,N^3^,N^3^-Tetramethyl-4-(5-nitrobenzo[c][1,2,5]oxadiazol-4-yl)benzene-1,3diamine* (**13**): eluent: ethyl acetate/dichloromethane 8/2; yield 53%; brown solid, m.p.: >155 °C (dec); IR (*v*, cm^−1^): 1602, 1490, 1289, 1236; ^1^H-NMR (CDCl_3_, 400 MHz, 25 °C): δ (ppm): 7.81 (d, *J* = 9.4 Hz, 1H); 7.77 (d, *J* = 9.4 Hz, 1H); 7.46 (d, *J* = 9.0 Hz, 1H); 6.52 (d, *J* = 9.0 Hz, 1H); 6.41 (br.s, 1H); 3.06 (s, 6H); 2.45 (br.s, 6H); ^13^C-NMR (CDCl_3_: 100.56 MHz, 25 °C): δ (ppm): 153.4; 152.5; 150.6; 149.2; 145.9; 132.1; 128.2; 126.4; 113.9; 112.3; 106.5; 103.1; 42.0; 40.3; ESI-MS (*m*/*z*): 328 [M + H]^+^, 350 [M + Na]^+^, 366 [M + K]^+^; HRMS (ES^+^) *m*/*z*: [M + H]^+^ calculated for C_16_H_18_N_5_O_3_ 328.1410 found 328.1411.

*5-(2,4-Di(piperidin-1-yl)phenyl)-4-nitrobenzo[c][1,2,5]oxadiazole* (**14**): eluent: ethyl acetate/dichloromethane 2/8; 38%; brown solid, m.p.: >120 °C (dec); IR (*v*, cm^−1^): 1598, 1506, 1319, 1235; ^1^H-NMR (CDCl_3_, 400 MHz, 25 °C): δ (ppm): 7.96 (d, *J* = 9.0 Hz, 1H); 7.67 (d, *J* = 9.0 Hz, 1H); 7.10 (br.s, 1H); 6.63 (br.s, 2H); 3.30 (br.s, 4H); 2.84 (br.s, 4H); 1.73 (br.s., 4H); 1.64 (br.s, 4H); 1.43 (br.s, 4H); ^13^C-NMR (CDCl_3_: 100.56 MHz, 25 °C): δ (ppm): 154.2; 153.5; 149.0; 144.2; 140.9; 137.2; 130.8; 122.5; 121.3; 118.7; 110.0; 106.1; 53.5; 49.3; 29.7; 25.9 (two signals overlapped); 24.1; ESI-MS (*m*/*z*): 408 [M + H]^+^, 430 [M + Na]^+^, 446 [M + K]^+^ HRMS (ES^+^) *m*/*z*: [M + H]^+^ calculated for C_22_H_26_N_5_O_3_ 408.2036 found 408.2033.

*5-(2,4-Di(pyrrolidin-1-yl)phenyl)-4-nitrobenzo[c][1,2,5]oxadiazole* (**16**): eluent: ethyl ether/petroleum light 7/3; yield 50%; orange solid, m.p.: >115 °C (dec); IR (*v*, cm^−1^): 1601, 1503, 1321, 1233; ^1^H-NMR (CDCl_3_, 400 MHz, 25 °C): δ (ppm): δ, ppm: 7.89 (d, *J* = 9.4 Hz, 1H); 7.62 (d, *J* = 9.4 Hz, 1H); 7.05 (d, *J* = 8.6 Hz, 1H); 6.34–6.25 (m; 2 H, two signals overlapped); 3.42 (t, *J* = 6.5 Hz, 4H), 3.16 (s; 2H); 3.04 (s, 2H) 2.08 (t, *J* = 6.48 Hz, 4H); 1.83 (t, *J* = 6.33 Hz, 4H); ^13^C-NMR (CDCl_3_: 100.56 MHz, 25 °C): δ (ppm): 149.4; 148.9; 144.3; 142.2; 136.5; 132.1; 118.9; 105.8; 100.8; 51.7; 50.0; 25.6; 25.2; (selected data); ESI-MS (*m*/*z*): 380 [M + H]^+^, 402 [M + Na]^+^; HRMS (ES^+^) *m*/*z*: [M + H]^+^ calculated for C_20_H_22_N_5_O_3_ 380.1723 found 380.1723.

*N^1^,N^1^,N^3^,N^3^-Tetramethyl-4-(4-nitrobenzo[c][1,2,5]oxadiazol-5-yl)benzene-1,3diamine* (**17**): eluent: ethyl ether/petroleum light 3/7;yield 36%; brown solid, m.p.: >130 °C (dec); IR (*v*, cm^−1^): 1604, 1498, 1315, 1237;^1^H-NMR (CDCl_3_, 400 MHz, 25 °C): δ (ppm): 77.94 (d, *J* = 9.4 Hz, 1H); 7.64 (d, *J* = 9.4 Hz, 1H), 7.11 (d, *J* = 8.6 Hz, 1H); 6.50 (br.s, 2H, two signals overlapped); 3.07 (s, 6H), 2.65 (s, 6H); ^13^C-NMR (CDCl_3_: 100.56 MHz, 25 °C): δ (ppm): 152.9; 152.1; 149.0; 148.9; 144.3; 140.8; 136.4; 132.8; 131.4; 127.5; 119.1; 107.7; 103.5; 43.3; 41.2; ESI-MS (*m*/*z*): 328 [M + H]^+^, 350 [M + Na]^+^, 366 [M + K]^+^; HRMS (ES^+^) *m*/*z*: [M + H]^+^ calculated for C_16_H_18_N_5_O_3_ 328.1410 found 328.1413.

*7-(2,4-Di(piperidin-1-yl)phenyl)-4,6-nitrobenzo[c][1,2,5]oxadiazole-1-oxide* (**19**): eluent: ethyl ether/petroleum light 1/1; yield 80%; brown solid, m.p.: >280 °C (dec); IR (*v*, cm^−1^): 1610, 1554, 1520, 1312;^1^H-NMR (CDCl_3_, 400 MHz, 25 °C): δ (ppm): 8.80 (s, 1H); 7.66 (d, *J* = 9.7 Hz, 2 H, two signals overlapped); 6.66 (br.s, 1H); 3.43–3.30 (m, 4H); 2.91–2.75 (m, 4H); 1.77 (br.s, 2H); 1.69 (s, 2H); 1.49 (br.s, 4H); ESI-MS (*m*/*z*): 469 [M + H]^+^, 491 [M + Na]^+^, 507 [M + K]^+^; HRMS (ES^+^) *m*/*z*: [M + H]^+^ calculated for C_22_H_25_N_6_O_6_ 469.1836 found 469.1838.

*7-(2,4-Di(morpholin-1-yl)phenyl)-4,6-nitrobenzo[c][1,2,5]oxadiazole-1-oxide* (**20**): eluent: ethyl acetate/*n*-hexane 7/3; yield 80%; brown solid, m.p.: >280 °C (dec); IR (*v*, cm^−1^): 1607, 1549, 1515, 1313; ^1^H-NMR (CDCl_3_, 400 MHz, 25 °C): δ (ppm): 8.77 (s, 1H); 7.00 (d, *J* = 9.0 Hz, 1H); 6.67 (dd, *J*_1_ = 8.5 Hz, *J*_2_ = 1.8 Hz, 1H); 6.62 (d, *J* = 1.85 Hz, 1H); 3.87 (t, *J* = 4.6 Hz, 4H); 3.50–3.39 (m, 4H); 3.35 (t, *J* = 4.6 Hz, 4H); 2.97–2.78 (m, 4H); ^13^C-NMR (CDCl_3_: 100.56 MHz, 25 °C): δ (ppm): 154.3; 153.9; 144.3; 142.1; 134.1; 133.9; 131.1; 127.7; 113.7; 111.8; 110.3; 105.5; 67.0; 66.3; 52.8; 47.8. ESI-MS (*m*/*z*): 473 [M + H]^+^, 495 [M + Na]^+^, 511 [M + K]^+^; HRMS (ES^+^) *m*/*z*: [M + H]^+^ calculated for C_20_H_21_N_6_O_8_ 473.1421 found 473.1423.

*7-(2,4-Di(pyrrolidin-1-yl)phenyl)-4,6-nitrobenzo[c][1,2,5]oxadiazole-1-ozide* (**21**): eluent: ethyl ether/petroleum light 1/1; yield 65%; brown solid, m.p.: >250 °C (dec); IR (*v*, cm^−1^): 1609, 1546, 1516, 1313; ^1^H-NMR (CDCl_3_, 400 MHz, 25 °C): δ (ppm): 8.85 (s, 1H); 7.01 (d, *J* = 8.7 Hz, 1H); 6.75 (d, *J* = 8.7 Hz, 1H); 6.72 (br.s, 1H); 3.68–3.59 (m, 4H); 4.48-3.41 (m, 4H); 2.17–2.05 (m, 8H); ESI-MS (*m*/*z*): 441 [M + H]^+^, 463 [M + Na]^+^; HRMS (ES^+^) *m*/*z*: [M + H]^+^ calculated for C_20_H_21_N_6_O_6_ 441.1523 found 441.1526.

*7-(2,4-Bis(dimethylamino)phenyl)-4,6-nitrobenzo[c][1,2,5]oxadiazole-1-ozide* (**22**): eluent: ethyl ether/petroleum light 8/2; yield 65% (70% in presence of Al_2_O_3_); brown solid, m.p.: >130 °C (dec); IR (*v*, cm^−1^): 1612, 1545, 1510, 1314; ^1^H-NMR (CDCl_3_, 400 MHz, 25 °C): δ (ppm): 8.88 (s, 1H); 7.66 (d, *J* = 8.9 Hz, 1H); 6.56 (dd, *J*_1_ = 8.8 Hz, *J*_2_ = 2.1 Hz, 1H); 6.35 (s, 1H); 3.14 (s, 6H), 2.54 (s, 6H); ^13^C-NMR (CDCl_3_: 100.56 MHz, 25 °C): δ (ppm): 154.1; 151.8; 143.2; 142.3; 134.2; 133.5; 131.4; 128.2; 111.3; 107.2; 102.4; 43.2; 40.2; ESI-MS (*m*/*z*): 389 [M + H]^+^, 411 [M + Na]^+^; HRMS (ES^+^) *m*/*z*: [M + H]^+^ calculated for C_16_H_17_N_6_O_6_ 389.1210 found 389.1211.

*4,6-Dinitro-7-(4-(pyrrolidin-1-yl)phenyl)benzo[c][1,2,5]oxadiazole 1-oxide* (**25**): eluent: petroleum light/diethyl ether 6/4; yield 66%, dark blue solid; m.p. > 280 °C (dec); IR (*v*, cm^−1^): 1614, 1556, 1522, 1312; ^1^H-NMR (CDCl_3_, 400 MHz, 25 °C): δ (ppm): 8.68 (s, 1H), 7.23 (d, *J* = 8.71 Hz, 2H), 6.63 (d, *J* = 8.71 Hz, 2H), 3.43 (t, *J* = 6.37 Hz, 4H), 2.08 (t, *J* = 6.37 Hz, 4H); ^13^C-NMR (CDCl_3_, 100.56 MHz) δ (ppm): 150.4, 147.7, 141.4, 134.6, 131.5 (CH), 127.9 (CH), 113.5, 112.3, 111.8 (CH), 110.9, 47.7 (CH_2_), 25.4 (CH_2_); ESI-MS (*m*/*z*): 372 [M + H]^+^, 394 [M + Na]^+^; HRMS (ES^+^) *m*/*z*: (M + H)^+^ calculated for C_16_H_14_N_5_O_6_ 372.0944 found 372.0946.

*7-(4-(Dimethylamino)phenyl)-4,6-dinitrobenzo[c][1,2,5]oxadiazole 1-oxide* (**26**): petroleum light/diethyl ether 4/6; yield 62% dark blue solid; m.p.: >145 °C (dec); IR (*v*, cm^−1^): 1610, 1554, 1521, 1309; ^1^H-NMR (CDCl_3_, 400 MHz, 25 °C): δ (ppm): 8.69 (s, 1H), 7.24 (d, *J* = 9.10 Hz, 2H), 6.76 (d, *J* = 9.10 Hz, 2H), 3.12 (s, 1H); ^13^C-NMR (CDCl_3_, 100.56 MHz) (selected data) δ (ppm): 152.7, 144.6, 131.2 (CH), 127.8 (CH), 111.5 (CH), 40.0 (CH_3_); ESI-MS (*m*/*z*): 346 [M + H]^+^, 368 [M + Na]^+^, 384 [M + K]^+^; HRMS (ES^+^) *m*/*z*: [M + H]^+^ calculated for C_14_H_12_N_5_O_6_ 346.0788 found 346.0790.

## 4. Conclusions

The nucleophile/electrophile combinations between 1,3-dialkylaminobenzene derivatives **1**–**4** and the chloro-nitrobenzofurazan derivatives **5**–**7** or the chloro-dinitrobenzofuroxan **18** occurred regioselectively, yielding the product from the attack in the less-hindered *ortho*-position to the amino groups located on the aromatic ring of the nucleophile. The novel synthesized compounds are highly conjugated systems contemporarily bearing an electron-rich and an electron-poor moiety. This characteristic feature makes them interesting candidates for eventual future applications in applied fields, such as solar cells, optoelectronic, and chromogenic materials. In the case of benzofuroxan derivatives, in the pharmaceutical field as NO release agents.

Further, the extension to some monoaminobenzene derivatives as nucleophiles permits to highlight the effect of steric encumbrance of the amino substituents on the reactivity of the considered systems on going from mono- to towards polysubstituted species.

## Figures and Tables

**Table 1:** Electrophile/nucleophile combinations ^1^ monitored via ^1^H-NMR ^2^.

Entry	Electrophile	Nucleophile	Solvent	Product	10 min	2 h	24 h	48 h	72 h
1	**5**	DPBH (**1**)	CDCl_3_	**8**	-	4	21	26	26 ^3^
2	**5**	DPBH (**1**)	CD_3_CN	**8**	9	12	48	50	52 ^4^
3	**5**	DNMeBH (**4**)	CDCl_3_	**11**	4	21	40	40	n.c. ^5^
4	**5**	DNMeBH (**4**)	CD_3_CN	**11**	25	65	73	76	n.c. ^5^
5	**5**	DPyBH (**3**)	CD_3_CN	**10**	42	53	53	56	n.c. ^5^
6	**7**	DPBH (**1**)	CD_3_CN	**14**	15	40	55	n.c.	60
7	**7**	DNMeBH (**4**)	CD_3_CN	**17**	16	40	55	n.c.	55
8	**7**	DPyBH (**3**)	CD_3_CN	**16**	35	55	63	70	n.c. ^5^
9	**18**	DPBH (**1**)	CD_3_CN	**19**	27	>98	/	/	/
10	**18**	DMBH (**2**)	CD_3_CN	**20**	25	87	95	>98	/
11	**18**	DNMeBH (**4**)	CD_3_CN	**22**	3	32	87	>98	/
12	**18**	DPyBH (**3**)	CD_3_CN	**21**	>97 ^6^	/	/	/	/

^1^ Reaction between equimolar amounts of the (*N*,*N*-dialkyl)-diaminobenzene (0.05 mmol) and benzofurazan- (or benzofuroxan-)derivative (0.05 mmol). The reagents were mixed in a vial and dissolved in 0.7 mL of deuterated solvent. ^2^ Relative % conversion was calculated from the ^1^ H-NMR spectrum with respect to the remained electrophile signals. ^3^ The conversion became 55% after 24 h from the addition of triethylamine. ^4^ After 24 h from the addition of triethylamine, the conversion became nearly quantitative. ^5^ n.c. means not calculated. ^6^ The spectrum showed the presence of other unidentified products.
